# Obituary

**DOI:** 10.21307/jofnem-2020-044

**Published:** 2020-04-28

**Authors:** Manzoor H. Soomro

**Affiliations:** Economic Cooperation Organization Science Foundation (ECOSF), 5 Floor MoST Building, 1-Constitution Avenue, G-5/2, Islamabad-44000, Pakistan

Shahina Fayyaz (1959–2020)

Professor Shahina Fayyaz, an eminent nematologist of Pakistan, passed away on March 20, 2020 in Karachi, Pakistan. She was suffering from a lung ailment for a few months.


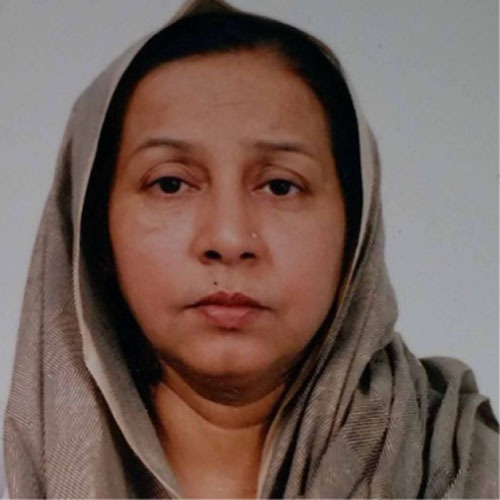


Professor Shahina Fayyaz was born on April 4, 1959 in Karachi, Pakistan. She served the National Nematological Research Centre (NNRC) for 38 years with utmost dedication and professionalism. She played a key role in developing and strengthening the NNRC, which led to its recognition by the FAO, UN as the Centre of Excellence in Nematode Taxonomy in the Near East Region in 1992. The Centre gained ISO Certification 9001:2015 for Diagnostic Services and Educational Programs in Nematology under her leadership.

Dr Shahina received her MSc (Plant Pathology) in 1980 from the Department of Botany, University of Karachi, Pakistan and joined the NNRC’s original team as a Research Fellow in 1981. She was appointed as Research Officer in the PL-480 project in 1982 and was selected as Scientific Officer in the Centre in 1988. She earned her PhD from the University of Karachi in 1989 under the supervision of Dr M.A. Maqbool. Shahina became Senior Scientific Officer in 2000 and began to lead the Centre as In-charge in 2002 after the retirement of her mentor/director, Dr Maqbool; she was appointed as Director of NNRC in 2003. In 2009, she was appointed as Full Professor and, in recognition of her contributions, she was elevated as Meritorious Professor in 2013. She retired as Director of NNRC and as an accomplished professional nematologist on April 3, 2019, although continued supervision of her PhD students till her death.

Dr Shahina Fayyaz established herself as a very accomplished researcher, and won and undertook 33 research projects as Principal Investigator (PI) (20 projects) and as Co-PI (13 projects) funded by various agencies such as International Foundation for Science, Pakistan Science Foundation (PSF), Pakistan Agricultural Research Council, Higher Education Commission of Pakistan, and World Wide Fund for Nature, Pakistan. She also won the European Commission’s post-doctoral fellowship for research at CAB International labs at St Albans in the United Kingdom. She pioneered research on entomopathogenic nematodes (EPN) in Pakistan and added a state of the art EPN laboratory at the NNRC. She obtained a US patent and 17 Pakistan national industrial patents on EPN techniques and EPN use as bio-pesticides. She published over 200 research papers in peer reviewed journals of international repute as well as numerous books, booklets, and proceedings. Shahina was the Secretary General of Pakistan Society of Nematologists (PSN) and Managing Editor of Pakistan Journal of Nematology published by PSN, till her departure. She was the winner of the competitive “Dr Z.A. Hashmi R&D Gold Medal” of PSF in 2013 and had served as Member of the Technical Committees and Board of Trustees of the Foundation.

Her rather early departure from this world at age of less than 61 years is a great loss for all of us − her friends, colleagues and students, farmers and anyone who has had the privilege of knowing her. But above all, the greatest loss to her husband and son, they can be reached on the E-mail: shahinafayyaz@gmail.com.

